# Neuroanatomical Basis of Individuality in Muscle Tuning Function: Neural Correlates of Muscle Tuning

**DOI:** 10.3389/fnbeh.2019.00028

**Published:** 2019-03-06

**Authors:** Kahori Kita, Rieko Osu, Chihiro Hosoda, Manabu Honda, Takashi Hanakawa, Jun Izawa

**Affiliations:** ^1^Center for Frontier Medical Engineering, Chiba University, Chiba, Japan; ^2^Advanced Telecommunications Research Institute International, Kyoto, Japan; ^3^Faculty of Human Sciences, Waseda University, Saitama, Japan; ^4^Graduate School of Arts and Sciences, University of Tokyo, Tokyo, Japan; ^5^Department of Functional Brain Research, National Center of Neurology and Psychiatry, Tokyo, Japan; ^6^Integrative Brain Imaging Center, National Center of Neurology and Psychiatry, Tokyo, Japan; ^7^Information and System, Faculty of Engineering, University of Tsukuba, Ibaraki, Japan

**Keywords:** muscle tuning function, gray matter volume, VBM, motor control, brain structure

## Abstract

In a conventional view of motor control, the human brain might employ an optimization principle that leads a stereotypical motor behavior which we observe as an averaged behavioral data over subjects. In this scenario, the inter-individual motor variability is considered as an observation noise. Here, we challenged this view. We considered a motor control task where the human participants manipulated arm force by coordinating shoulder and elbow torques and investigated the muscle-tuning function that represents how the brain distributed the ideal joint torques to multiple muscles. In the experimental data, we observed large inter-individual variability in the profile of a muscle-tuning function. This contradicts with a well-established optimization theory that is based on minimization of muscle energy consumption and minimization of motor variability. We then hypothesized the inter-subject differences in the structure of the motor cortical areas might be the source of the across-subjects variability of the motor behavior. This was supported by a voxel-based morphometry analysis of magnetic resonance imaging; The inter-individual variability of the muscle tuning profile was correlated with that of the gray matter volume in the premotor cortex which is ipsilateral to the used arm (i.e., right hemisphere for the right arm). This study suggests that motor individuality may originate from inter-individual variation in the cortical structure.

## Introduction

A theory of motor control postulates that, in all individuals, the brain employs a certain principle that produces stereotypical motor behavior to achieve a given task. However, the data from individuals often deviate slightly from the ideal motor trajectory predicted by a theoretical model, even though the averaged trajectory fits very well with the model prediction. This inter-individual variability in motor behaviors contradicts the theory; however, the cause of this diversity in movement trajectory has not yet been examined in depth. Here, we aim to explore a source of the inter-individual variability of a motor behavior to reexamine a general theoretical framework.

The contemporary consensus in the motor control community regarding the mechanisms of how the brain solves this redundancy problem is that the brain may employ an optimization principle that minimizes the cost and maximizes the task performance (Todorov and Jordan, 2002; Izawa and Shadmehr, 2008, 2011) to keep consistence of intra-individual motor behavior. Because different types of cost functions that include different state variables (e.g., muscle force or joint torques) predict dissociable motor behaviors, we are able to infer a type of cost function that the brain might adopt. To date, ample evidence has suggested that both the motor costs that penalize energy consumptions (Emken et al., 2007; Izawa and Shadmehr, 2008, 2011) and the accuracy costs that penalize the influence of motor noise (Harris and Wolpert, 1998) on the precision of motor movement are essential components of the optimization principle the human brain employs (O'Sullivan et al., 2009).

However, in these previous studies of motor control, scientists paid little attention to the effect of the limited neural resources in computing motor commands. The structure of the brain that characterizes neural resources for the brain to optimize motor commands to achieve the certain task requirement might alter the generated motor behaviors if this variation of the motor commands does not interfere with the task performance.

**Figure 1:**
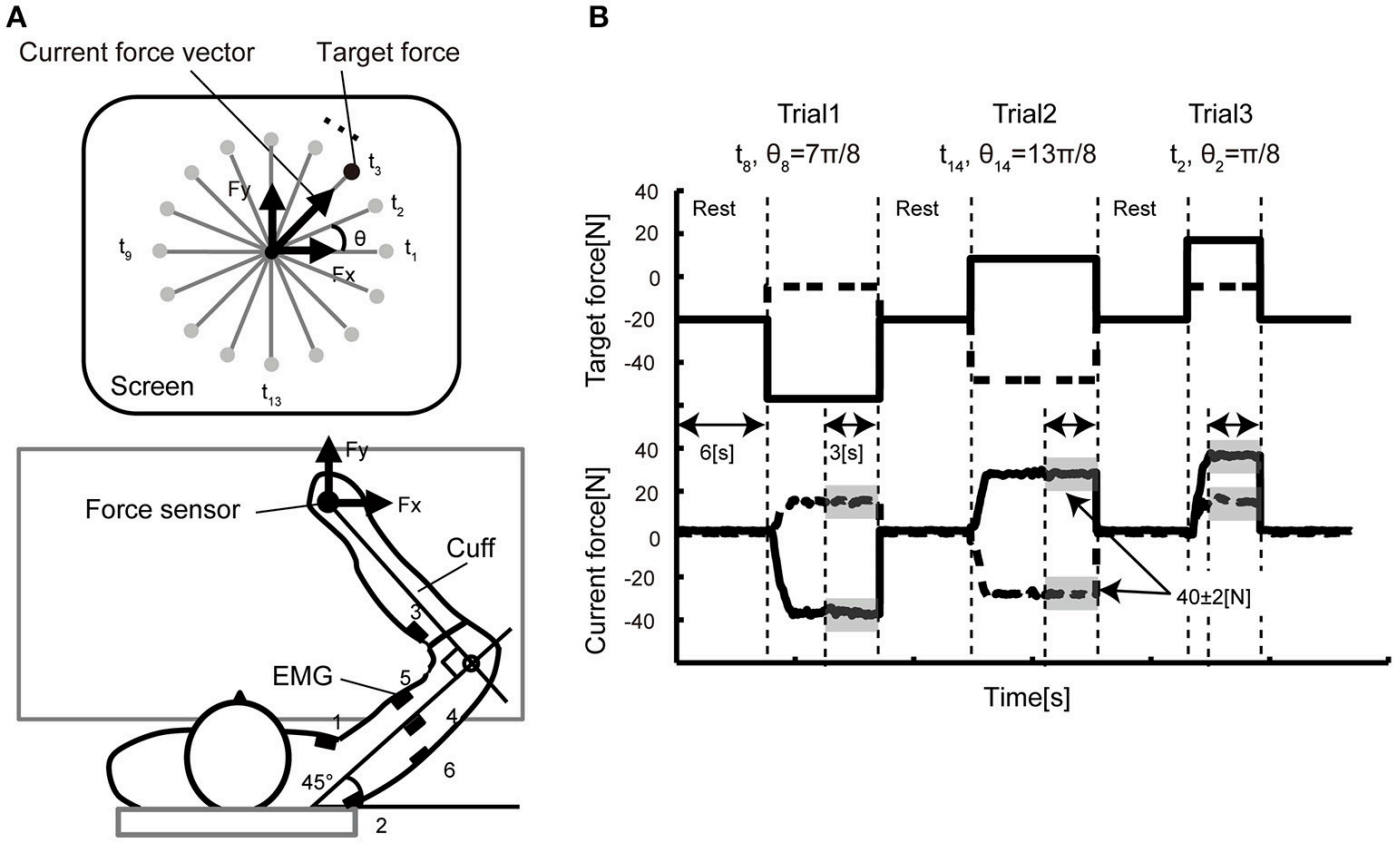
Experimental setup. **(A)** In the isometric force production task, the participants held the handle of a force sensor mounted on a horizontal table, and they were asked to manipulate the cursor presented on a vertical LCD monitor. The monitor indicated the force vector detected from the sensor. In each trial, a force target was randomly selected from 16 directions positioned every 22.5 degrees. The coordinate system of the vertical display and the arm forces were defined **(A)** such that the forward direction in the task space corresponded to the upward direction (y-axis) in the display space. EMG signals were recorded from six electrodes attached over the six different muscles (1: pectoralis major, 2: posterior deltoid, 3: brachioradialis, 4: lateral head of the triceps brachii, 5: biceps brachii, 6: long head of the triceps brachii) of the right arm. **(B)** The time course of the target presentation. The top graph shows the target force required for successful reaching over time. The solid line represents the x-axis component and the dashed line the y-axis component. The bottom graph shows the example data of the force sensor measured from a representative participant. After a 6-s rest period, a randomly selected target appeared on the screen. As soon as the participants noticed the target, they pushed the handle such that the force cursor could reach the target. When the cursor position was held inside the target area represented by the gray shadow (± 2N in size) for 3 s, the trial ended and moved on to the next trial.

To test this idea, we employed a well-established force control task where a participant manipulated arm force in the horizontal plane, by coordinating torques of both the elbow and the shoulder joints (Figure 1A). In this scenario, the brain has to solve the redundancy problem in order to coordinate the activations of at least six muscles that produce these joint torques. Here, we specifically hypothesized that the structure the cortical motor area might influence the coordination of multiple muscles, yielding a correlation between the inter-individual variability of the muscle-tuning function and the inter-individual variability of the density of the neuronal population. To test this hypothesis, we analyzed anatomical MRI data obtained from all participants who underwent the hand-force control task. Next, we conducted voxel-based morphometry (VBM) analysis to explore whether a cluster of voxels in the motor cortex might explain the variability of the motor behaviors.

## Materials and Methods

### Participants

We measured EMG data during an isometric force production task and obtained T1-weighted images from all participants. Thirty volunteers without any history of the neurological disorder participated in these experiments (all males; ages 21–27 years; all right-handed). All participants gave written informed consent before participating in the study. The experimental paradigm was approved by the local ethics committee of the National Institute of Neuroscience, National Center of Neurology and Psychiatry and Tokyo Bay Rehabilitation Hospital and was conducted according to the Declaration of Helsinki.

### Experimental Design and Data Acquisition

Figure 1 shows the experimental setup of the behavioral experiment. The participants held the handle of the force sensor by the right hand and were instructed to produce joint torques. The wrist joint of the participant was fixed by the cuff, and only shoulder and elbow joint rotations in the horizontal plane were permitted. The cuff was tightly coupled to the handle, and the upper arm of the participants was supported in by the table. The participants were able to control their hand force by the torque produced from the only right elbow and shoulder joints. We asked the participants to put their left hand on a left thigh and relaxed.

EMG signals were recorded using an amplifier (The Bagnoli 8 EMG System, DELSYS). EMG Sensors were placed over the major muscles for flexion and extension of shoulder and elbow in the right side; the shoulder monoarticular flexor (pectoralis major) and extensor (posterior deltoid), an elbow monoarticular flexor (brachioradialis) and extensor (lateral head of triceps brachii) and a biarticular flexor (biceps) and extensor (long head of triceps brachii). EMG Signals were sampled with 16-bit A/D acquisition systems at a sampling rate of 2000 Hz and analyzed offline. The amplifier was set to a gain 1000 and a range of ±5 V.

In the experiment, the participants were instructed to produce 40 N by applying force to the handle of a force sensor in 16 directions (*θ*_1_, …, *θ*_16_ = 0, π/8, π/4, …, 15π/8 rad) in the hand's x–y plane. The current force vector (Fx, Fy) and a small red circle indicating target direction (*t*_1_, …, *t*_16_) of each trial were displayed on the computer monitor. The participants were required to keep 40 ± 2 N for 3 s for each trial. The participants conducted 10 trials for each target; 160 trials in total. The participants took a 6-s rest between each trial and a 1- to 10-min rest per 16 trials to avoid muscle fatigue.

High-resolution T1-weighted anatomical images were obtained with magnetization prepared rapid gradient-echo images (TR = 2000 ms; TE = 4.4 ms; FA = 80; matrix = 192 × 176; FOV = 192 × 176; voxel size 1 × 1 × 1 mm^3^) on a 3-T MRI scanner (Siemens Trio, Erlangen, Germany) with an 8-channel phased array receiver-only coil.

### Data Analysis

#### EMG Data Pre-processing

Each EMG signal was band-pass filtered from 5 −500 Hz and rectified. We calculated the average of the EMG during a holding period of all trials (10 trials) for each target (*t* = 1, …,16), and then the EMGs were normalized in each muscle (*i* = 1, …,6).

(1) $$\begin{array}{r} u_{i}^{t}=\frac{e_{i}^{t}-min_{i}}{max_{i}-min_{i}} \end{array}$$

where *e* is the average EMG, min_*i*_ and max_*i*_ are minimum and maximum amplitude in each muscle and $u_{i}^{t}$ becomes 1 or 0 in the direction of the largest and smallest muscle activation, respectively. The $u_{i}^{t}$ shows the tuning function of each muscle.

To quantify the broadness of the muscle-tuning function, we computed the summation of the normalized muscle activities, $u_{i}^{t}$, over all target directions *t* across six muscles:

(2) $$\begin{array}{r} \left[broadness\left(a.u.\right)\right]=\overset{6}{\underset{i=1}{\sum }}\overset{16}{\underset{t=1}{\sum }}u_{i}^{t} \end{array}$$

which is identical to the total area surrounded by the muscle-tuning functions.

#### MRI Data Pre-processing and VBM

The high-resolution 3D T1-weighted images were subjected to a VBM analysis using VBM8 toolbox (Ashburner and Friston, 2000) implemented in SPM8 (Wellcome Trust Center of Neuroimaging, UCL). The preprocessing steps were as follows. First, each image was segmented into gray matter, white matter and cerebrospinal fluid in the native image space. Second, the gray matter images were transformed into the Montreal Neurological (MNI) space. Third, these normalized gray matter images were smoothed using a Gaussian kernel of 10 mm full-width at half-maximum. We investigated the correlation of broadness of the tuning function with regional gray matter volume. A voxel-wise multiple regression was conducted, using the tuning function as an independent variable and biomechanical factors such as arm length and weight as confounding covariates. The search volume was limited to the right or left primary motor cortex (Brodmann area 4) and right or left premotor and supplementary motor areas (Brodmann area 6). The voxelwise significance level was set at *p* < 0.05, corrected for multiple comparisons in terms of the familywise error (FWE) rate.

## Results

### Tuning Functions Computed From EMG Signals

The participants were asked to hold a handle mounted on a table with their right hand, and to produce the required force vector by pushing the handle (Figure 1A). After a 6-s rest period, a reaching target was shown on a vertical screen. The participants were asked to move a cursor to the reaching target immediately after the target was displayed. The participants then continued to apply arm force to keep the cursor inside the target for 3 s. The movement of the cursor reflected the direction/size of the arm forces in the horizontal plane. A single trial ended when the cursor was held inside the target for 3 s. The participants were requested to keep 40 ± 2 N for 3 s for each trial. A single trial lasted until the produced force satisfied this condition. Figure 1B illustrates the example of the sequence of the target force on X and Y axis with a representative force data.

**Figure 2:**
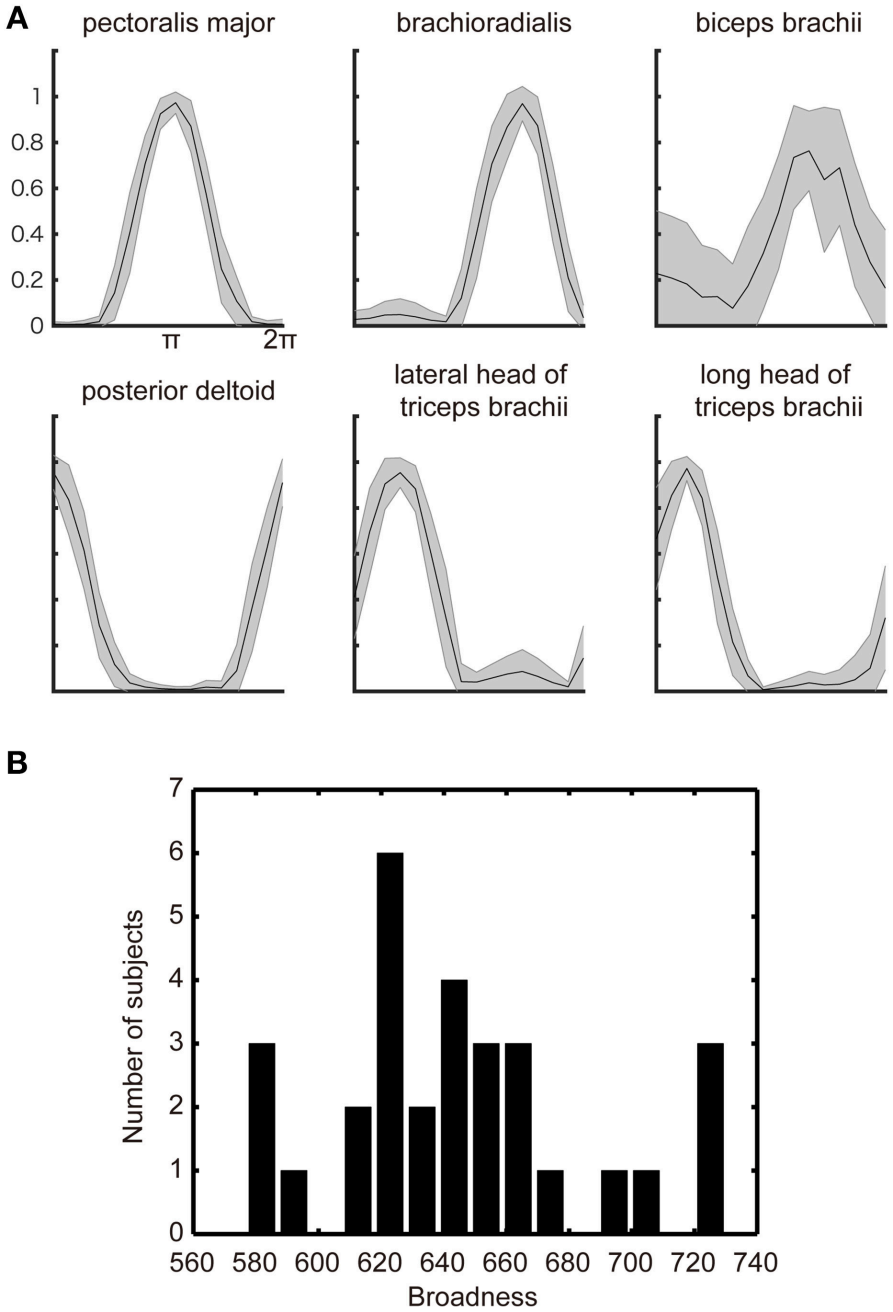
Muscle-tuning function. **(A)** The across subjects average of the normalized EMG signal with STD (gray shadow) measured from electrodes attached over the six representative muscles of the right upper limb. The data were averaged for each target direction and plotted after normalized by the maximum EMG across all targets. **(B)** Histograms of the individual differences in the broadness of the muscle-tuning function. The broadness (arbitrary unit) was calculated as the area surrounded by the tuning function of each muscle and summed across all muscles in each participant (See section Materials and methods). The histogram was plotted from all participants' EMG data.

Figure 2A shows the subjects across average of the normalized EMG signals over the target angle measured from the electrodes attached over the six muscles of the right arm of a representative participant. Additionally, as has been observed in the previous studies (Hoffman and Strick, 1999; van Bolhuis and Gielen, 1999; Nozaki et al., 2005; Kurtzer et al., 2006), each muscle shows a unimodal tuning function with a specific preferred direction. These observed properties are congruent with the previous model of muscle recruitment where the muscle activation is optimized to minimize the muscle energy consumption, in addition to minimizing the force vector error (van Bolhuis and Gielen, 1999; Fagg et al., 2002; Kurtzer et al., 2006).

The optimization principle predicts a stereotypical muscle tuning function no matter which optimization principle adopted by the model. However, the participants' actual behaviors showed the large inter-individual variability in the broadness of the muscle-tuning function as replicated in the present study (Figure 2B). We thus explored the neurophysiological correlates of the inter-individual variability in the broadness of the tuning function.

### Voxel-Based Morphometry

**Figure 3:**
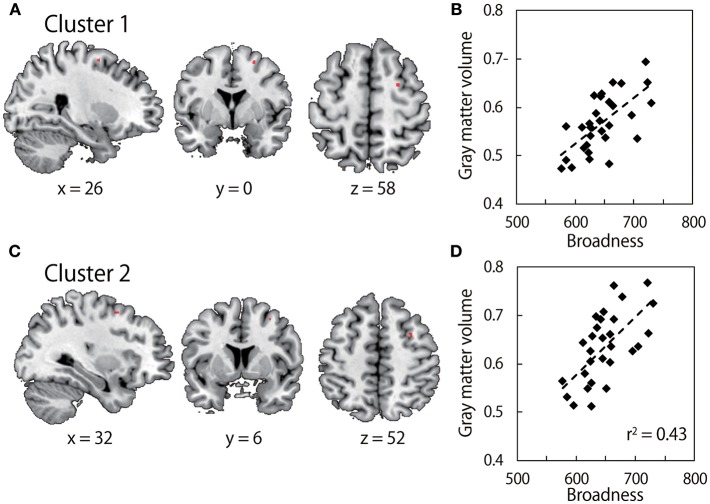
Results of Voxel-based morphometry (VBM). **(A,C)** show gray matter volume which significantly correlates with the broadness of the muscle tuning function. The colored area in each brain image indicates clusters after family-wise error (FWE) correction for multiple voxels in volume of interest (Brodmann Aera) with a threshold of *P* < 0.05 at the cluster level. Survived two clusters (cluster 1: MNI coordinate *x* = 26, *y* = 0, *z* = 58; cluster size, 7 voxels, cluster 2: MNI coordinates, *x* = 32, *y* = 6, *z* = 52; cluster size, 4 voxels) are shown in upper images and lower images, respectively. **(B,D)** show correlation between C*beta values within the clusters in left panels, i.e., gray matter volume, and the broadness of the muscle tuning function.

Our hypothesis here is that the inter-individual variability of the neural structure might explain the inter-individual variability of the broadness of the muscle-tuning function. We analyzed anatomical MRI obtained from all participants who performed the arm force task using a VBM analysis. We sought clusters of voxels in the motor-related cortical areas that explain the inter-individual variability of the broadness of the tuning function. Figure 3 illustrates that the gray matter volume in the region of right dorsal premotor cortex was significantly correlated with the broadness of the muscle-tuning function (Cluster 1at the MNI coordinate of x = 26, y = 0, z = 58, P = 0.040 FWE corrected, 7 voxels; Cluster 2 at x = 32, y = 6, z = 52, P = 0.043 FWE corrected, 4 voxels).

## Discussion

Studies of motor control have strongly relied on the hypothesis that the brain might employ a common cost function across all human participants to make a motor plan for a certain task. Whereas the stereotypical motor behaviors such as smooth reach trajectory (Abend et al., 1982; Flash and Hogan, 1985) or distributed muscle forces (Ashburner and Friston, 2000) have been found in human motor behavior, a large inter-individual variability around the stereotypical movement was also evident. Here, we exemplified the inter-individual variability in the muscle force tuning function when the participants controlled arm force and sought the source of the individuality of the motor behaviors. We found that the broadness of the tuning function had large variability across participants. This result suggests that the process of optimization for a certain cost function is influenced by an intrinsic property of the individual participant. To explore the neurophysiological correlates of this phenomenon, we conducted VBM on the anatomical MRI data taken from the same participants who participated in the behavioral study. We found that the gray matter volume of the ipsilateral side to the used arm of the motor-related area was significantly correlated with the broadness of the muscle-tuning function. It is unlikely that the VBM findings reflect other biomechanical confounding factors (arm length and weight) than the muscle tuning function since these variables were included as covariates in the GLM.

We observed an influence of the premotor area of the right hemisphere on shaping muscle tuning function while it is not clear how the right hemisphere shape the motor commands formed in the left motor cortex. A study with transcranial magnetic stimulation showed that a conditioning stimulus over right premotor cortex induced suppression of the motor-evoked potentials of the left motor cortex, suggesting interhemispheric inhibition via commissural fibers between the premotor cortex and the contralateral motor cortex (Mochizuki et al., 2004). A similar interhemispheric interaction has been found in another transcranial magnetic stimulation study between the primary motor cortex and the ventral premotor cortex (Baumer et al., 2009). This interhemispheric inhibition might lead to broader muscle-tuning functions. However, evidence of interhemispheric inhibition does not reject the other possibility of the descending pathway from the right premotor area (Benecke et al., 1991; Galea and Darian-Smith, 1994; Staudt et al., 2002). While the projection from the right hemisphere to the right arm is a minor pathway in terms of its number of neurons, it has the capacity to explain the observed correlation between the gray matter volume around the right premotor area and the broadness of the muscle-tuning function in a more straightforward way. We propose that further studies should be conducted with diffusion tensor axonal tracking techniques (Lehericy et al., 2004; Wahl et al., 2007) to examine ipsi/contralateral hemispheric contributions to corticomuscular optimization.

One of the limitations of this study is not taking the previous history of arm training into consideration. In fact, the prior history of the practice of motor control increases the accuracy of the motor skills, which is mediated by the structural plasticity associated with the long-range practice and corresponding neural activity change (Dayan and Cohen, 2011). This long-range training induces axon sprouting, dendritic branching, neurogenesis, glial changes, and angiogenesis, which all leads the gray matter increase (Zatorre et al., 2012). Thus, the volume of the gray matter of the individual subject is determined by the history of the training, which is captured by the anatomical MRI and VBM analysis. Each subject must have a unique history of motor training, and thus the variety of experience, such as tennis, golf, or running, of all participants, is too large to standardize this experience. We rather think that the gray matter volume is the standardized index which captures the property of the motor skill at the moment of the experiment. Our aim here is to seek the anatomical feature that causes a difference in the tuning function which may be affected by the history of the training.

Another limitation of this study is that any neural activity was not examined. As we discussed above, the gray matter volume change may lead neural activity change. Indeed, the limitation of our study is that any neural activity was measured while the subject is controlling the hand force. The signals measured in fMRI might contain richer information of how the motor-related are in the cortex activated differently among the subjects. However, at the same time, the influence of the history of the motor experience on the fMRI result is complex with respect to the gray matter volume change. For instance, fMRI signal (BOLD signal) is influenced by both the short-range experience and the long-range experience of the motor training and the decrease of the BOLD due to the short range experience is also observed (Dayan and Cohen, 2011) We considered that while how the brain optimize neural activity is influenced by many different factors such as metabolism or short range experience, this optimization process is always constrained by the cortical structure which leads variation in motor behavior. Thus, the specific aim of this paper is to examine how the long-range factors which change the structure of the cortex alter how the nervous system optimize the muscle force distribution problem.

A potential problem of this analysis is a spurious correlation. We report here that we found two clusters which are correlated with the broadness of the muscle tuning function. These two clusters lie close each other (10 voxels apart), and both are in the right premotor cortex. While spurious correlation can't be rejected ultimately in all VBM analysis in general, the SPM is implemented with multiple approaches to minimize to detect spurious correlation (Wilke and Lidzba, 2007). As it is suggested in the standard procedure of the SPM package, we used the strict thresholding by using the FWE correction. In addition, evaluating the cluster size is reported as the effective approach (Wilke and Lidzba, 2007). According to these criteria, one of the clusters of the two (cluster size n = 7) is above minimum cluster size and is able to be considered as a meaningful cluster. While the ipsilateral (i.e., right hemisphere) influence on the right limb movement is counter-intuitive, a number of studies reported the contribution of the motor is in the right hemisphere to the right limb control.

It puzzled us that we did not detect any significant correlation between the left premotor cortex involved in computing motor commands to control isometric force on the right arm. Indeed, the task performance improvement achieved by the long-range training with arm's reach task increased the gray matter volume of the left motor cortex (Landi et al., 2011). While a further investigation with the isometric force control task of the present paper is certainly necessary, the previously reported change of the gray matter volume in the left motor cortex and the left premotor cortex is not necessary to guarantee the influence of the gray matter volume in the left motor cortices on the tuning function of the muscle force production during the isometric force production task. One important difference between the previous report and our study is that we focused on the performance irrelevant feature of the muscle activities whereas most of the previous report discussed the correlation between the performance (i.e., accuracy) and the cortical structure. The optimization of the muscle tuning function involves the highly complicated computational processes (Fagg et al., 2002). In fact, the gray matter volume change in the right premotor cortex without the change in the left premotor cortex was reported for the complex force production task (Gryga et al., 2012). We speculate that, whereas the contralateral contribution is dominant in skill formation in the task-relevant performance, the ipsilateral contribution is important in motor coordination in the task-irrelevant dimension.

There is also a number of specific limitations in this study. First, we recruited very specific cohort: male, young and right-handed. The goal of this study is to find a correction between the neural structure (GLM) which is influenced by the long-range experience of the limb control and the tuning function of the EMG . Theoretically, the tuning function is determined by solving the muscle force distribution problem and, in this computational process, not only the neural structure, but biomechanical factors also constrain the optimization process. In addition, age and sex-difference altered GLM (Pfefferbaum et al., 1992; Chen et al., 2007). In order to reduce the effects of these factors and to see the effect of the long-range experience of the limb control dominantly, we recruited only male, young and right-handed subjects. Second, we did not assess the tuning function of the distal hand muscles and finger muscles. As described in the method section, we constrained the subject's hand by the cuff which is tightly connected to the handle of the force sensor. This cuff prevents the subject form holding the handle while pushing the force sensor. Thus, the distal hand and the finger muscles were not requested to achieve tasks. This technique has been repeatedly used in the field of motor control when we would like to minimize the effect of the distal hand force and finger muscles when we measure limbs stiffness which is potentially influenced by the distal hand motion (Gomi and Kawato, 1996). Lastly, we analyzed only gray matter volume and did not examine other potential correlates such as the diffusion tensor imaging and the resting state functional connectivity (Kaiser et al., 2015; Song et al., 2015). Considering a process for the brain to solve the present task, the brain should use the entire cortical network with perceiving the target position first, computing the force vector second, and then solving the muscle force distribution problem. Thus, the long-range cortico-cortical connection and how the neural network taking advantage of this connection should influence the result of the optimization. It is reasonable to think that the tuning function might be influenced by the individuality of the anatomical and functional connectivity which are measured by the DTI fiber tracking method and by the resting state functional connectivity. we remain these studies for future work.

## Data Availability

The datasets generated for this study are available on request to the corresponding author.

## Author Contributions

KK and JI designed the study. KK collected both behavioral and MR data. CH helped to collect MR data. KK and JI analyzed data. KK and JI wrote the first draft of the paper. TH advised MRI data analysis. All authors revised the manuscript.

### Conflict of Interest Statement

The authors declare that the research was conducted in the absence of any commercial or financial relationships that could be construed as a potential conflict of interest.
